# Identification of candidate aberrant differentially methylated/expressed genes in asthma

**DOI:** 10.1186/s13223-022-00744-5

**Published:** 2022-12-22

**Authors:** Zongling Wang, Lizhi Wang, Lina Dai, Yanan Wang, Erhong Li, Shuyuan An, Fengliang Wang, Dan Liu, Wen Pan

**Affiliations:** 1 Department of internal medicine, Qingdao Fuwai Cardiovascular Hospital, 18th Floor north, 201 Nanjing Road, 266034 Qingdao, Shandong China; 2 Clinical laboratory, Qingdao Fuwai Cardiovascular Hospital, Qingdao, China

**Keywords:** Asthma, DNA methylation, Differentially expressed genes, Diagnosis, Bioinformatics analysis

## Abstract

**Background:**

Asthma is an important non-communicable disease worldwide. DNA methylation is associated with the occurrence and development of asthma. We are aimed at assuring differential expressed genes (DEGs) modified by aberrantly methylated genes (DMGs) and pathways related to asthma by integrating bioinformatics analysis.

**Methods:**

One mRNA dataset (GSE64913) and one gene methylation dataset (GSE137716) were selected from the Gene Expression Omnibus (GEO) database. Functional enrichment analysis was performed using GeneCodies 4.0 database. All gene expression matrices were analyzed by Gene set enrichment analysis (GSEA) software. STRING was applied to construct a protein-protein interaction (PPI) network to find the hub genes. Then, electronic validation was performed to verify the hub genes, followed by the evaluation of diagnostic value. Eventually, quantitative real-time polymerase chain reaction (qRT-PCR) was utilized to detect the expression of hub genes.

**Results:**

In total, 14 hypomethylated/high-expression genes and 10 hypermethylated/low-expression genes were obtained in asthma. Among them, 10 hub genes were identified in the PPI network. Functional analysis demonstrated that the differentially methylated/expressed genes were primarily associated with the lung development, cytosol and protein binding. Notably, HLA-DOA was enriched in asthma. FKBP5, WNT5A, TM4SF1, PDK4, EPAS1 and GMPR had potential diagnostic value for asthma.

**Conclusion:**

The project explored the pathogenesis of asthma, which may provide a research basis for the prediction and the drug development of asthma.

## Background

Asthma is a heterogeneous disease affecting people all over the world, characterized by chronic inflammation of the airway [1, 2]. It has a substantial impact on quality of life for many people [3]. Around 300 million people suffer from asthma, and it is likely that by 2025 a further 100 million may be influenced [3]. It is demonstrated that asthma is a complicated multifactorial disease whose etiology is attributed to interactions between genetic susceptibility, host factors and environmental exposures [3]. The mechanisms of asthma include airway inflammation, control of airway tone and reactivity [3, 4]. However, the underlying pathogenesis of asthma is poorly understood.

Epigenetics is the heritable alteration of gene expression unrelated to changes in DNA sequence [5]. Epigenetics is the link between environmental factors and genetic susceptibility, causing the disorder of development and function of the body’s immune system via affecting gene modification and regulating the function and characteristics of genetic genes [6, 7]. DNA methylation is the earliest and most significant modification in epigenetic regulation, which is involved in the complex interaction between gene and environment, playing a vital role in the occurrence of asthma [8]. Recent findings demonstrate that the environment and underlying genetic sequence variation could influence DNA methylation, which seems to modify the risk conferred by genetic variants for various phenotypes of asthma [8]. Nevertheless, it is still difficult to determine the certain genes and pathways.

High-throughput transcriptome integration analysis is able to collect the analysis results of related studies published on the same issue in order to obtain more accurate outcomes. It can produce significantly differentially expressed genes (DEGs) to avoid the inaccuracies of single research. Among all kinds of omics techniques, transcriptomics is the first developed and most widely applied. Microarray is the earliest and most widely utilized in transcriptome research and becomes a stable and reliable experimental technique. The development and application of microarray based on high-throughput sequencing may set the stage for the comprehensive study of the pathogenesis of asthma.

In this study, one gene expression dataset (GSE34913) and one gene methylation dataset (GSE137716) were obtained from Gene Expression Omnibus (GEO) to identify differentially methylated genes (DMGs) and DEGs. Various biological information databases were used to perform functional annotation of the DEGs, mining biological information under the microarray and screening out the key genes and crucial signaling pathways that affect the occurrence and development of asthma. Our study may enhance the understanding and development in regard to the gene expression of asthma.

## Methods

### Microarray data

The datasets were retrieved from GEO dataset by searching keywords “Asthma” and “Homo sapiens” [porgn: txid9606]. Datasets whose type were “Expression profiling by array” and “Methylation profiling by array” and meet the following criteria were included in this study: (1) the selected datasets must be genome-wide mRNA transcriptome data and DNA methylation data; (2) the data were obtained from airway epithelial samples of asthma and normal controls; (3) standardized or original datasets. The gene expression microarray (GSE64193) and the gene methylation microarray (GSE137716) were obtained. In total, 28 cases and 42 normal controls were included in GSE64193. The platform of the gene expression dataset was GPL570 [HG-U133_Plus_2] Affymetix human genome U133 Plus 2.0 array. For the gene methylation microarray, GSE137716 consisted of 9 cases and 7 normal controls. The gene methylation dataset used the platform GPL3534 Illumina HumanMethylation450 BeadChip (HumanMethylation450_15017482).

### Data acquisition and processing

Probes were mapped to genes, and the mean value of multiple probes corresponding to one gene was taken as the expression level of the gene. Limma package of software R-4.0.5 was utilized to perform the differential analysis for mRNA expression. P-Value < 0.05 and |log_2_fold change (FC)| > 0.5 were used as the cut-off standards to obtain DEGs. CHAMP was utilized to perform the differentially methylated analysis for the methylated dataset. P-Value < 0.05 and |Δβ| > 0.1 were set as screening criteria to obtain DMGs. Eventually, we intersected the DEGs and genes in the DMGs to obtain the hypermethylated/low-expression genes and hypomethylated/high-expression genes.

### Functional analysis

Functional enrichment analysis of Gene ontology (GO) and Kyoto Encyclopedia of Genes and Genomes (KEGG) was conducted on differentially methylated/expressed genes via GeneCodis4.0 database. The screening standard was false discovery rate (FDR) < 0.05. In addition, setting *P*-value < 0.05 as screening criterion, we performed GSEA on all genes expression matrices.

### Analysis of protein-protein interaction (PPI) network

For the purpose of investigating the protein interaction between the selected differentially methylated/expressed genes, we utilized the STRING database to construct a PPI network. Interaction score of 0.4 was considered as the cut-off criterion. Then, hub genes in the PPI network were filtered via CytoHubba plug-in. The intersection of the top 10 genes identified by MCC (Maximal Clique Centrality), MNC (Maximum Neighborhood Component), Degree, EPC (Edge Percolated Component), and closeness algorithms was selected and sorted, and 10 hub genes were finally selected.

### ROC analysis and and expression validation of hub genes

ROC curves of differentially methylated/expressed hub genes were plotted with pROC package and area under curve (AUC) of each curve was calculated to evaluate the diagnostic value. In addition, GSE85567 dataset was downloaded from GEO for conducting electronic validation of differentially methylated/expressed hub genes.

### Quantitative real-time polymerase chain reaction (qRT-PCR)

A total of 30 human blood samples (13 cases and 17 normal controls) were included in this study. some DEGs were selected as candidate genes for the detection. GAPDH and ACTB were used as reference genes. RNA samples were extracted from the collected samples. The primers of the target gene and the reference gene were utilized for amplification. The melting curve analysis was simultaneously conducted at 60–95℃. Amplification products were analyzed by 1.5% agarose RNA electrophoresis. Samples were then screened out to perform qRT-PCR assay on the basis of the results of agarose electrophoresis. Eventually, according to the raw results, relative quantitative results of target genes were calculated on the basis of the formula of 2^−△△ct^.

## Results

### Screening of differentially methylated/expressed genes in asthma

**Fig. 1:**
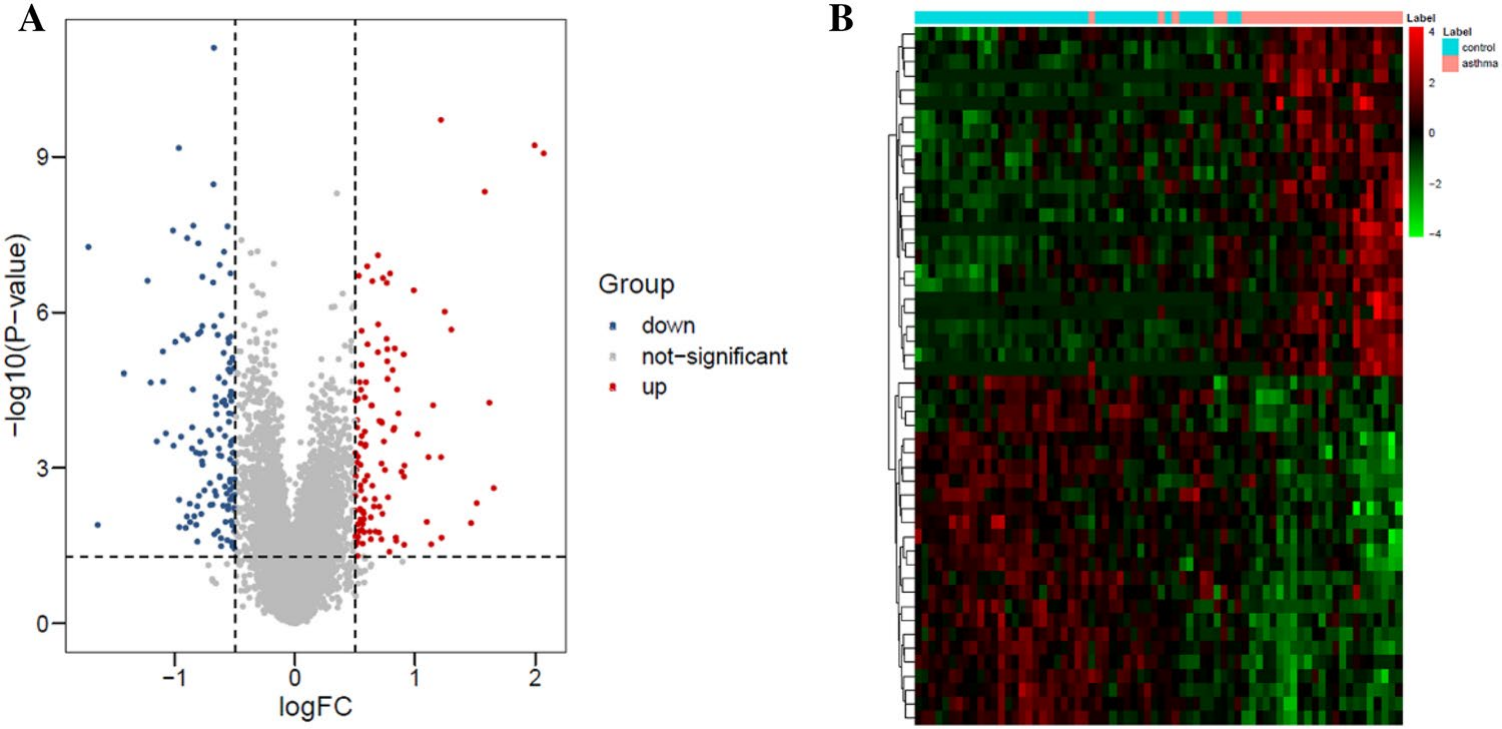
Analysis of DEGs in GSE64913 dataset. **A** the volcano plot of 245 DEGs; **B** the heat map of the DEGs.

**Fig. 2:**
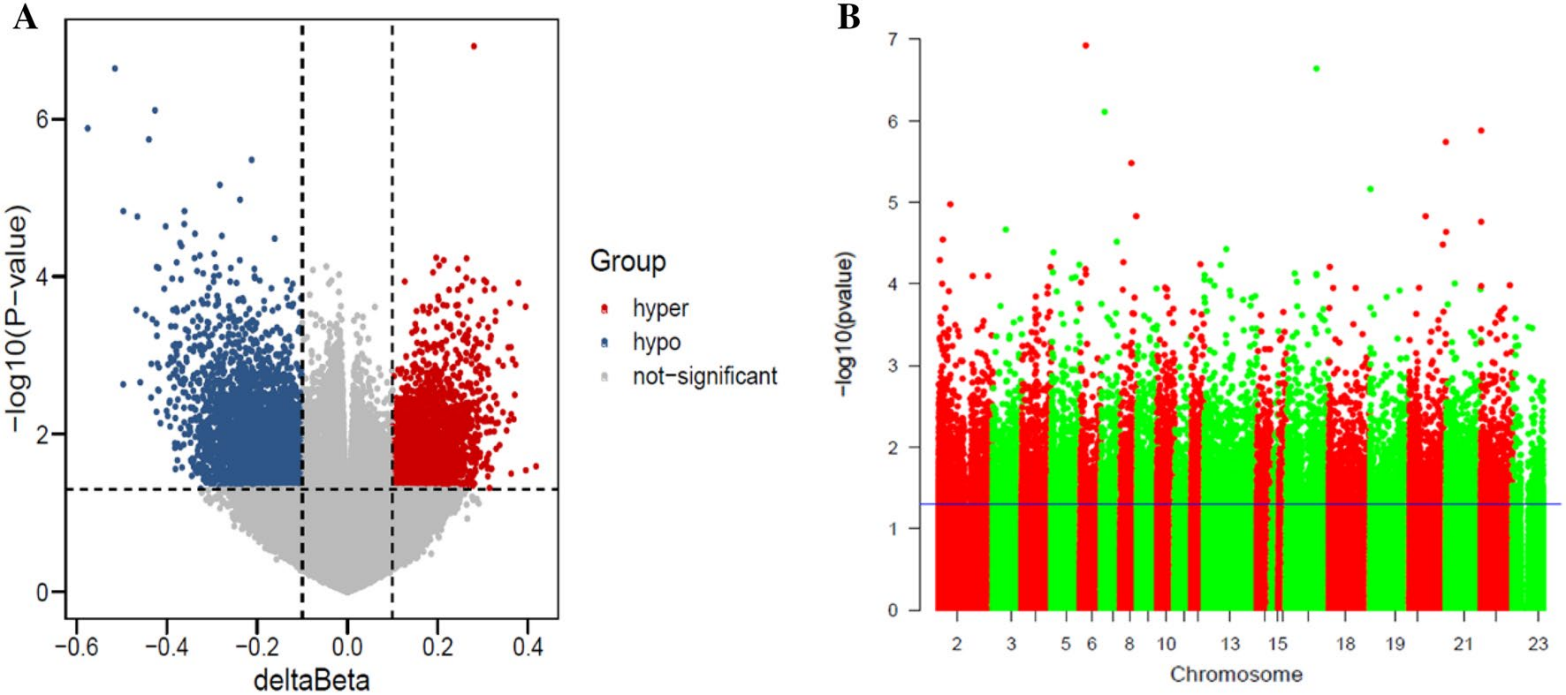
Analysis of DMGs in GSE137716 dataset. **A** The volcanic map of 3494 DMGs; **B** the Manhattan map of the 3494 DMGs.

**Fig. 3:**
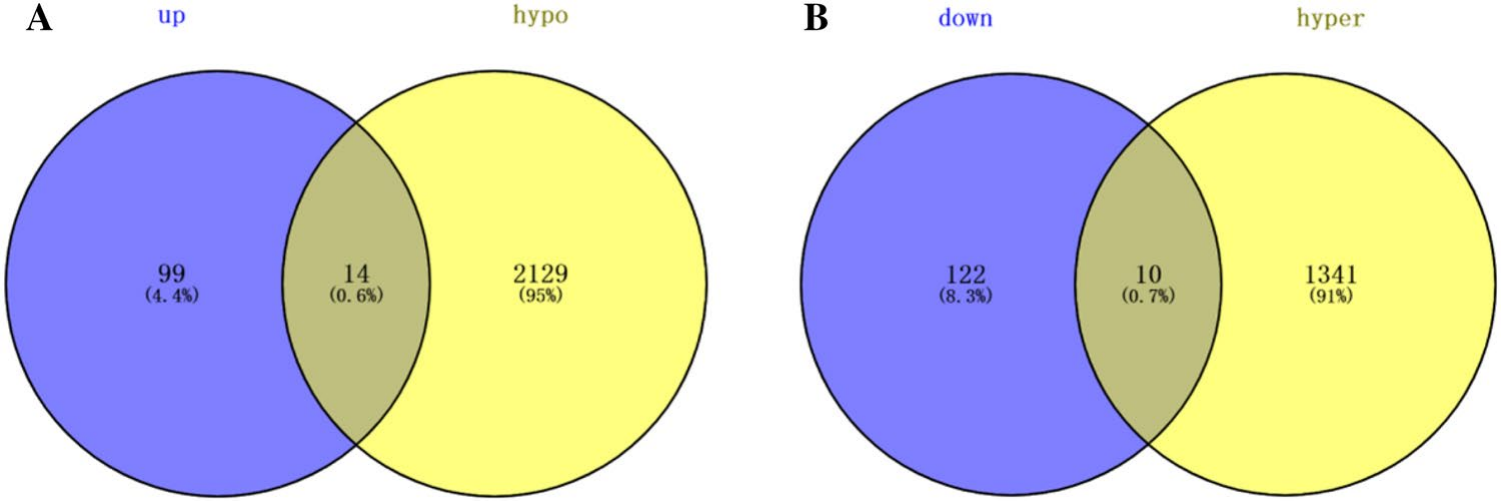
Differentially methylated/expressed genes. **A** Hypomethylated and up-regulated genes; **B** hypermethylated and down-regulated genes

Totally, 245 DEGs were identified after differential analysis, including 113 up-regulated and 132 down-regulated genes. The volcano plot and heat maps of 245 DEGs were shown in Fig. 1. A total of 8447 differentially methylated sites and 3494 DMGs were obtained, including 1351 hypermethylated genes and 2143 hypomethylated genes. Volcanic and Manhattan maps of 3494 DMGs are elucidated in Fig. 2. Then, through the intersection of 1351 hypermethylated genes and 132 down-regulated genes, 10 hypermethylated/down-regulated genes were obtained, including GRP, KCNA1, WNT5A, HLA-DOA, STEAP2, EPAS1, SLC23A1, MSMB, WIF1, and IFIT1. In addition, 14 hypomethylation/upregulated genes were obtained through the intersection of 2143 hypomethylation genes and 113 up-regulated genes, including FKBP5, CD109, CEACAM5, PDK4, PTPRH, TM4SF1, HN1, ZBTB16, POSTN, MX2, GMPR, C16orf54, CCL26, and NR4A3 (Fig. 3).

### GO and KEGG enrichment analysis

**Fig. 4:**
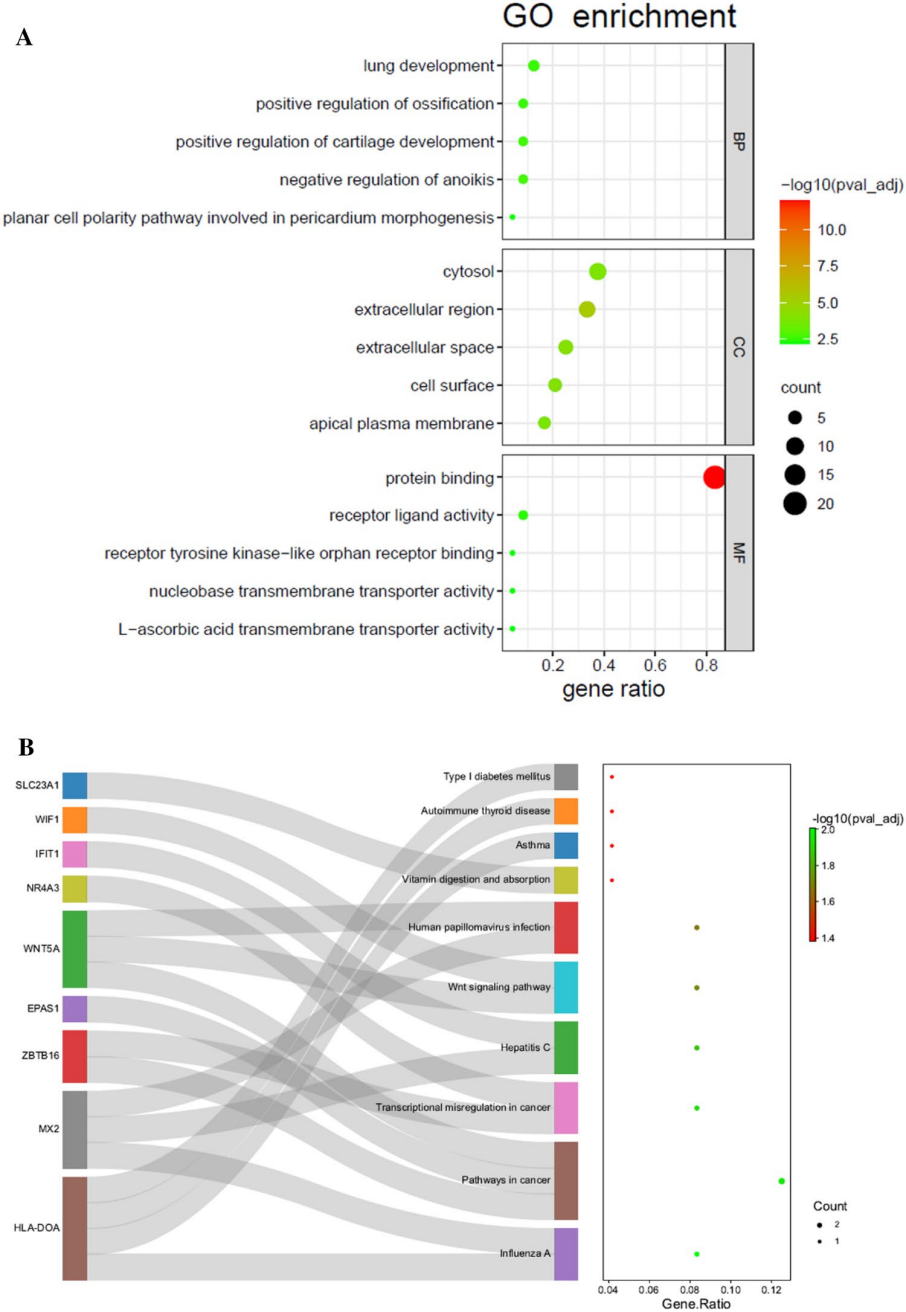
The functional enrichment analysis of all the differentially methylated/expressed genes. **A** The GO annotation. *BP*  biological process, *CC* cellular component, *MF* molecular function; **B** the KEGG of differentially methylated/genes

GO enrichment analysis showed that the differentially methylated/expressed genes are portrayed in Fig. 4 A. For biological process (BP), the genes were mainly linked to the lung development, positive regulation of ossification, positive regulation of cartilage development and negative regulation of anoikis. The cellular component (CC) enrichment analysis demonstrated that cytosol, extracellular region, extracellular space and cell surface were correlated with the genes. The molecular function (MF) analysis exhibited that the genes were mainly enriched in protein binding, receptor ligand activity and receptor tyrosine kinase-like orphan receptor binding. The KEGG pathway analysis showed that the analyzed genes were primarily linked to influenza A, pathways in cancer, transcriptional misregulation in cancer, hepatitis C, wnt signaling pathway, human papillomavirus infection, asthma, autoimmune thyroid disease and type I diabetes mellitus (Fig. 4B).

### GSEA analysis

**Fig. 5:**
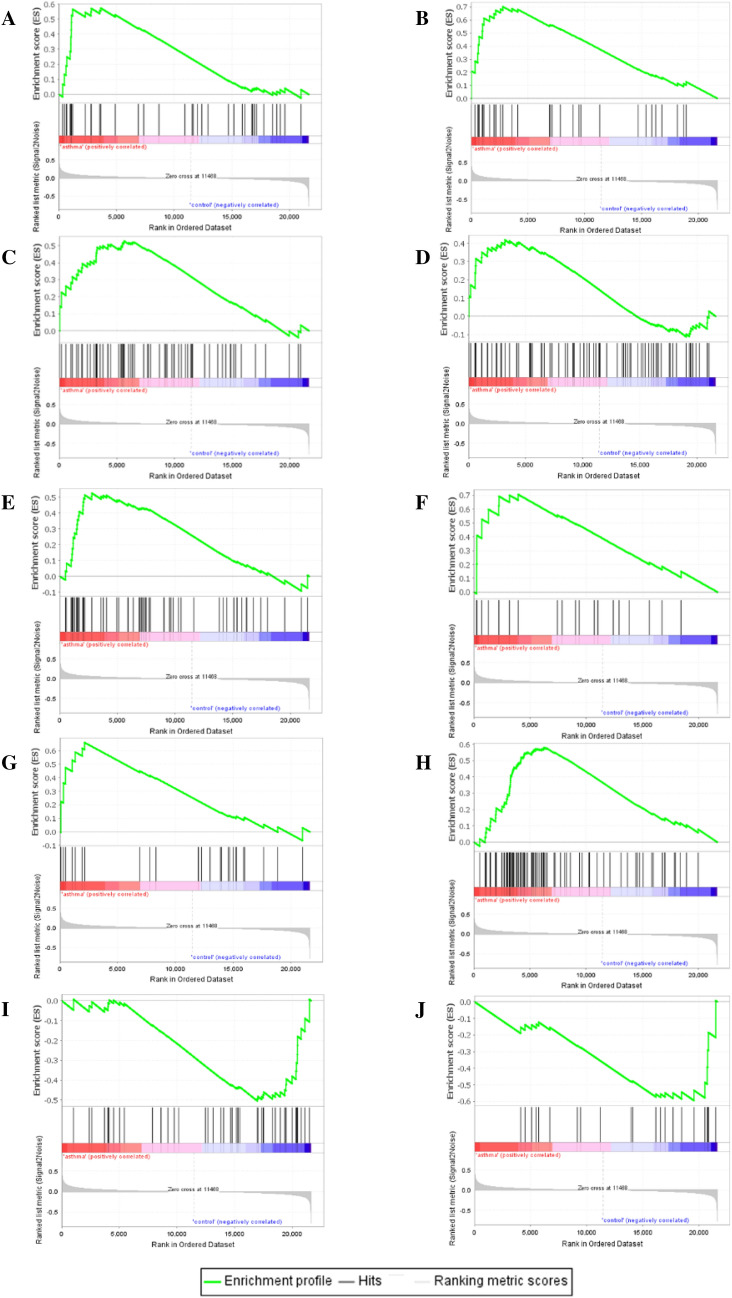
The GSEA of all genes. **A**–**H** Up-regulated pathways in the asthma group; **I**-**J** down-regulated pathways in the asthma group

We finally obtained eight significantly up-regulated gene sets in asthma, including AMINO SUGAR AND NUCLEOTIDE SUGAR METABOLISM, FRUCTOSE AND MANNOSE METABOLISM, CARDIAC MUSCLE CONTRACTION, HYPERTROPHIC CARDIOMYOPATHY HCM, GLYCOLYSIS GLUCONEOGENESIS, GLYCOSAMINOGLYCAN DEGRADATION, LIPID METABOLISM and PARKINSONS DISEASE (Fig. 5A-H). Two gene sets, NOTCH SIGNALING PATHWAY and DORSO VENTRAL AXIS FORMATION, were down-regulated in asthma (Fig. 5I, J).

### Construction of PPI networks

**Fig. 6:**
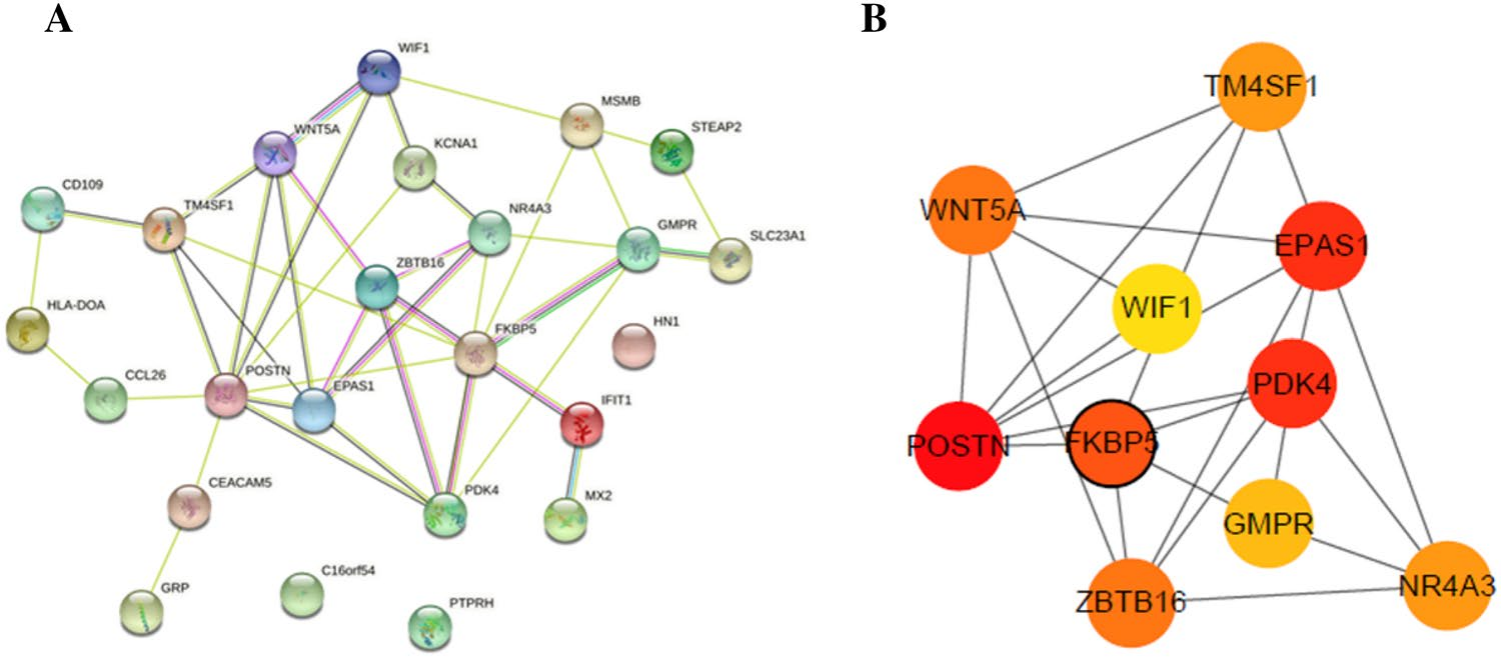
PPI networks of 24 differentially methylated/expressed genes and 10 hub genes. **A** PPI network of differentially methylated/expressed genes; **B** PPI network of 10 hub genes. The darker the color is, the more important it is. Nodes with a black border were DEGs derived from top 10 DEGs.

The PPI network (including 40 interacting gene pairs) obtained was presented in Fig. 6. Top 10 hub genes, consisting of POSTN, PDK4, EPAS1, FKBP5, ZBTB16, WNT5A, TM4SF1, NR4A3, GMPR and WIF1, were screened by the cytoHubba in Cytoscape software using five algorithms (Table 1). Hub genes in the PPI network are elucidated in Fig. 6. Among which, the top 3 hub genes were POSTN, PDK4 and EPAS1. FKBP5 was the only one gene that belongs to the top 10 up-regulated DEGs.

**Table 1:** Hub genes identified via five algorithms

Gene	MCC	MNC	Degree	EPC	Closeness
POSTN	18	7	9	12.973	14
PDK4	16	6	6	12.679	12.16667
EPAS1	16	6	6	12.606	11.66667
FKBP5	11	6	7	12.701	13.16667
ZBTB16	10	5	5	12.316	11
WNT5A	10	5	5	12.247	11.16667
TM4SF1	9	4	5	11.962	11.83333
NR4A3	9	4	5	12.111	10.91667
GMPR	7	4	5	11.73	11.16667
WIF1	5	3	4	11.341	10.91667

*MCC* maximal clique centrality, *MNC* maximum neighborhood component, *EPC* edge percolated component

### ROC analysis and expression validation of hub genes

**Fig. 7:**
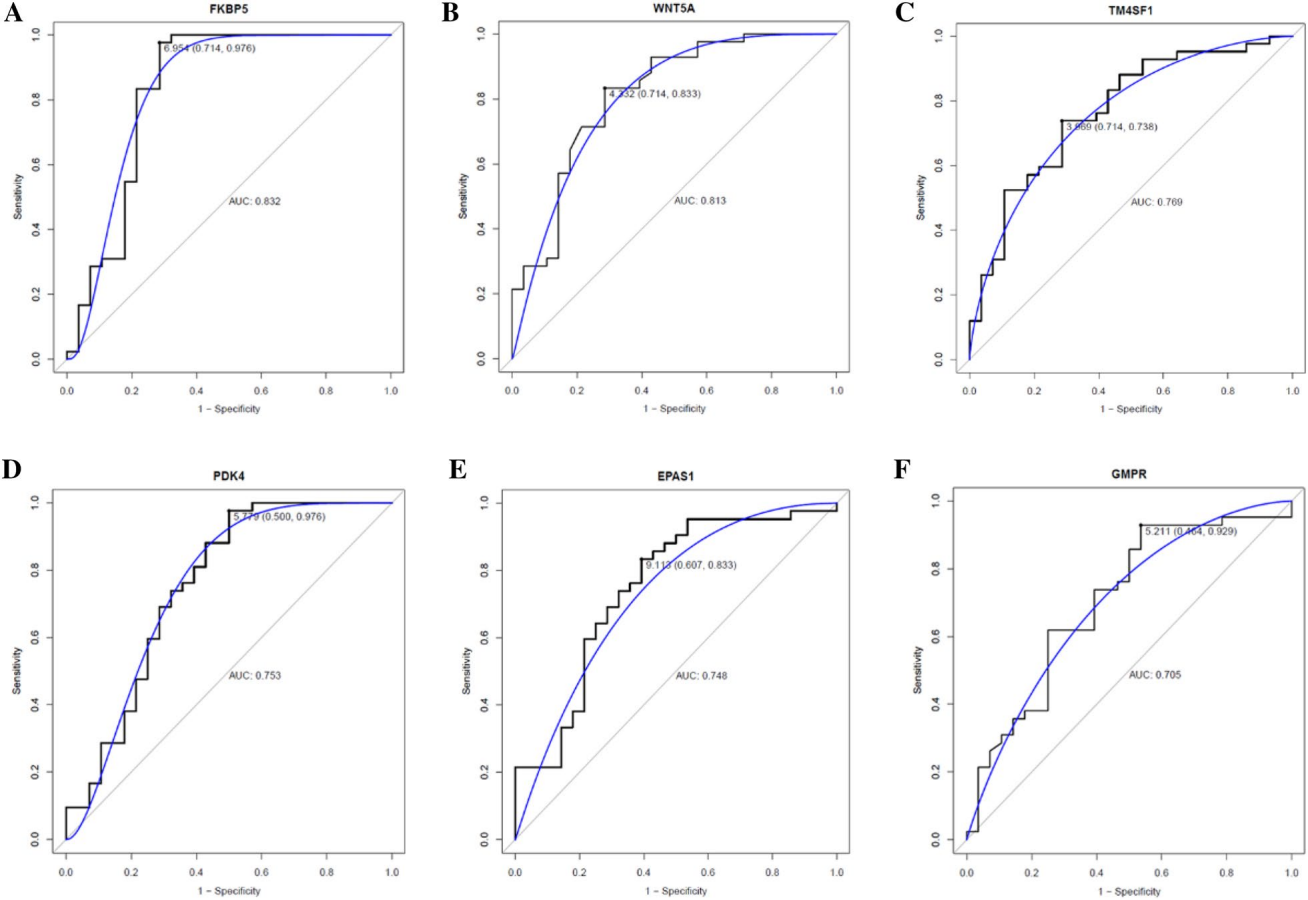
ROC curves of FKBP5 **A**, WNT5A **B**, TM4SF1 **C**, PDK4 **D**, EPAS1 **E** and GMPR **F**

**Fig. 8:**
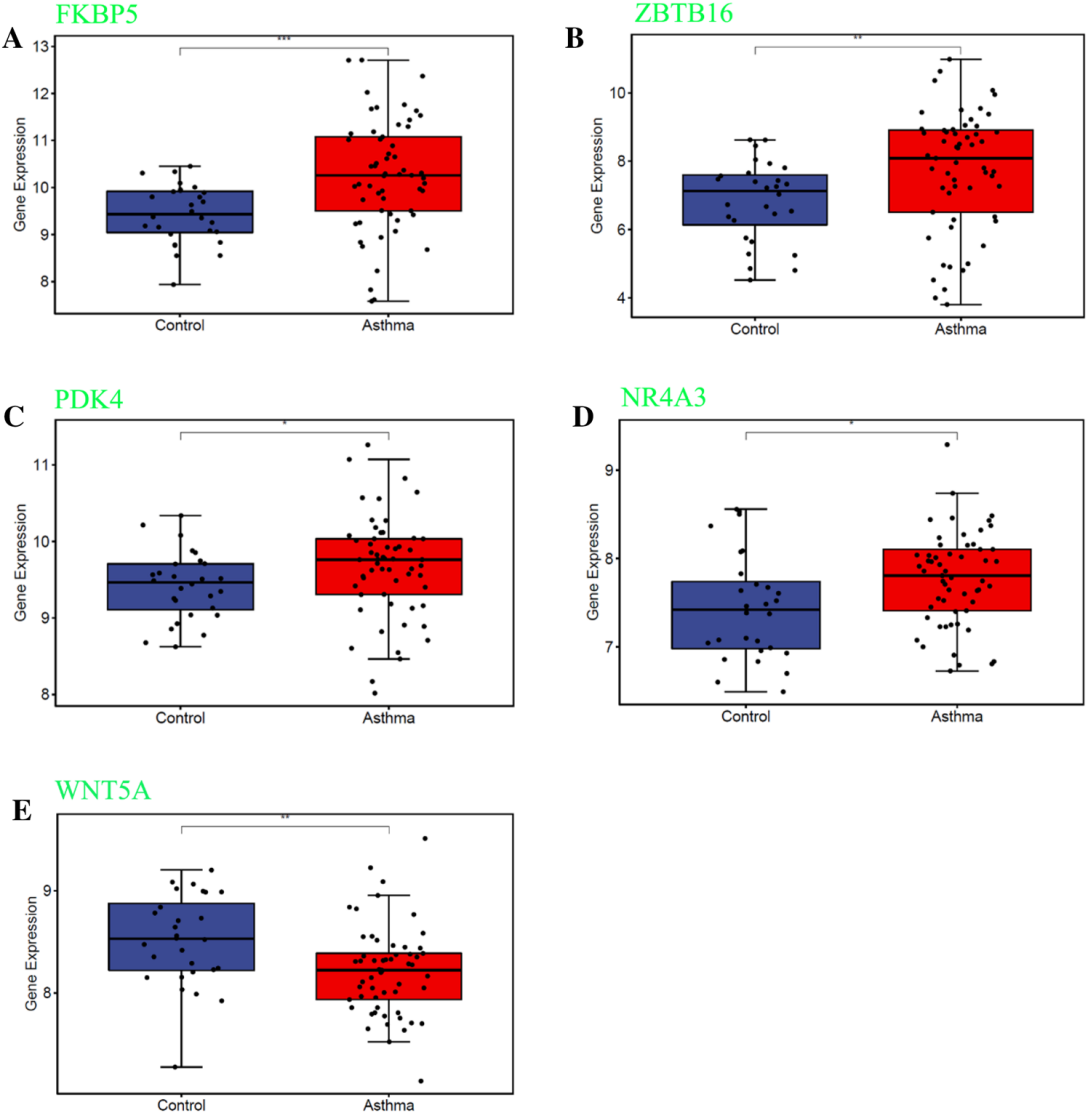
Expression validation of FKBP5 **A**, ZBTB16 **B**, PDK4 **C**, NR4A3 **D** and WNT5A **E** in GSE85567 dataset

The ROC curves of 6 differentially methylated/expressed hub genes are displayed in Fig. 7. The AUC value of FKBP5 (AUC = 0.832), WNT5A (AUC = 0.813), TM4SF1 (AUC = 0.769), PDK4 (AUC = 0.753), EPAS1 (AUC = 0.748) and GMPR (AUC = 0.705) was more than 0.7, which suggesting higher diagnostic value for asthma. Additionally, GSE85567 dataset was used for electronic expression validation of FKBP5, ZBTB16, PDK4, NR4A3 and WNT5A (Fig. 8). Expression trends of these genes were in line with our study.

### Detection of RT-qPCR

**Fig. 9:**
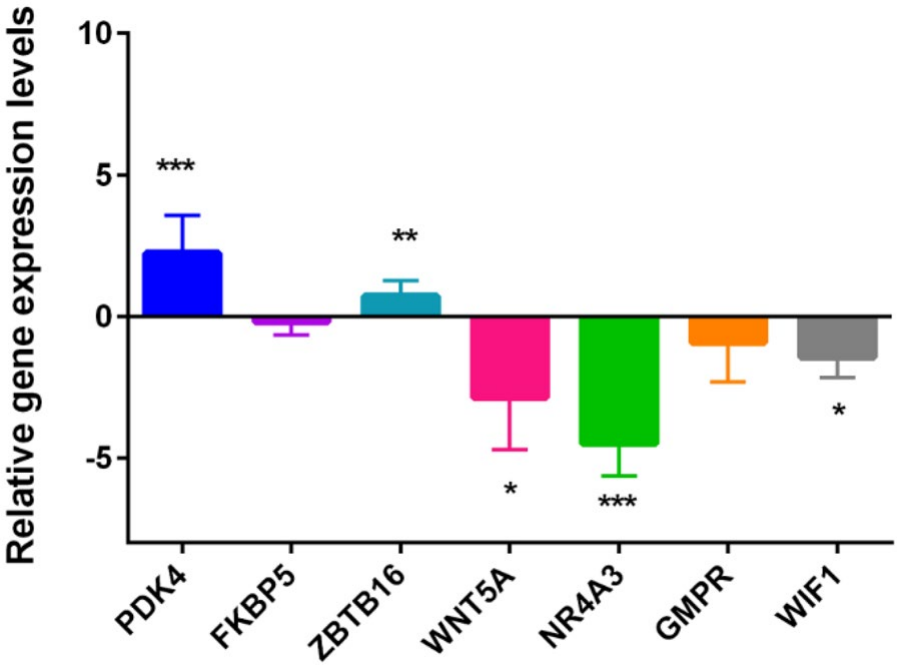
RT-qPCR validation of PDK4, ZBTB16, WNT5A, NR4A3 and WIF1. “^*^” represents *P*-value < 0.05; “^**^” represents *P*-value < 0.01; “^***^” represented *P*-value < 0.001

As illustrated in Fig. 9, PDK4 (*P*-value < 0.001) and ZBTB16 (*P*-value < 0.01) were up-regulated in asthma group, while WNT5A (*P*-value < 0.05), NR4A3 (*P*-value < 0.001) and WIF1 (*P*-value < 0.05) were down-regulated. The FKBP5 and GMPR did not show statistically significant.

## Discussion

Analysis of DEGs related to disease based on gene database has become a hot research modality in modern biomedicine [9]. In the current study, we identified the DEGs and DMGs in asthma compared with normal controls on the basis of high-throughput transcriptome integration analysis. The genes that were both hypermethylated/low-expression and hypomethylated/high-expression were obtained. We filtered and verified several hub genes and candidate genes related to asthma as well.

GO enrichment analysis of the differentially methylated/expressed genes was mainly enriched in the biological processes of lung development, positive regulation of ossification, positive regulation of cartilage development, negative regulation of anoikis and planar cell polarity pathway involved in pericardium morphogenesis. The lung development starts at 4 weeks of gestation and continues into early childhood, and bronchopulmonary dysplasia is attributed to the inadequate development of bronchial and lung [10]. Extensive researches of lung development have been conducted, generating new insights into the derivations of the different cell lineages existing in the lung and the molecular pathways that regulate these lineages, which contribute to the novel understandings of acquired lung diseases including asthma and COPD [11]. Hence, the outcome is consistent with our study of the GO enrichment. In addition, the CC analysis showed that these differentially methylated/expressed genes were primarily involved in cytosol, extracellular region, extracellular space, cell surface and the apical plasma membrane. Protein binding, receptor ligand activity, receptor tyrosine kinase-like orphan receptor binding, nucleobase transmenbrane transporter activity and L-ascorbic acid transmembrane transporter activity were significantly enriched molecular functions. Significantly enriched KEGG pathways included influenza A, pathways in cancer, and transcriptional misregulation in cancer. Notably, HLA-DOA, a hypermethylated/low-expression gene, was only one gene that enriched in the pathway of asthma. HLA-DO is a non-classical class II heterodimer consisting of ɑ and β chains, which are encoded by the HLA-DOA and HLA-DOB genes located in the HLA class-II region of MHC [12–14]. In the previous study, HLA-DOA genes were found a remarkable association with increased risk of diisocyanate-induced asthma [13], which revealed the closely correlation between HLA-DOA and asthma and provided a promising direction of the pathogenesis of asthma.

The analysis of GSEA found that the genes were mostly enriched in metabolism-related pathways. Among which, glucose metabolism, linoleic acid, arachidonic acid, nucleotide metabolism, energy metabolism and a variety of amino acids metabolism have been reported the significant differences existing between asthma patients and healthy controls [15–17].

The construction of PPI network of differentially methylated/expressed genes offered a method of identifying their functional connection, Cytoscape plugin cytoHubba provides 11 topological analysis methods including Degree, Edge Percolated Component (EPC), Maximum Neighborhood Component (MNC), Density of Maximum Neighborhood Component (DMNC), Maximal Clique Centrality (MCC) and six centralities (Bottleneck, EcCentricity, Closeness, Radiality, Betweenness, and Stress) based on shortest paths. There are no obvious advantages and disadvantages among algorithms, and different algorithms focus on different topological features. In general, MCC has proved to be a more accurate method for predicting important targets [18], MNC can be applied to discover some unrecognized hubs from previous dataset [19], Degree can be used to predict key proteins, proteins with a high Degree were more likely to be key proteins[20], EPC was used to explore protein interaction networks [21], Closeness was a topology analysis method based on shortest path [22]. In the previous research, MCC, MNC, Degree, EPC, and closeness algorithms were more commonly used [23, 24]. In this study, the above five algorithms were used to screen out the top 10 hub genes. Among them, PDK4, FKBP5, ZBTB16, WNT5A, GMPR and WIF1 are reported to be associated with asthma in the previous researches. NR4A3 is linked to the pulmonary vascular remolding. The ROC curves illustrated that FKBP5, WNT5A, PDK4, and GMPR had potential diagnostic value for asthma.

Pyruvate dehydrogenase kinase 4 (PDK4), one of four PDK isoenzymes expressed in a tissue-specific manner in mammals, is an important mitochondrial matrix enzyme in cellular energy regulation [25]. PDK4 is the main isoform in tissues demanding high energy, such as heart, skeletal muscle, lactating mammary gland, liver and vascular tissue [25–27]. Lee et al. found that PDK4 was up-regulated in the calcified vessels of atherosclerosis patients [27]. The data of researches demonstrated that PDK4 was a novel modulator of the integrity of the mitochondria-associated ER membrane (MAM) [25, 28–30]. New evidence confirmed that the repressive effects of the cornerstone of treatment for asthma, glucocorticoids, linked to the inflammatory pathways and genes and PDK4 can regulate glucose metabolism to explain the metabolic effects of glucocorticoids [31].

FK506-binding protein 5(FKBP5) is a 51-kDa protein with a C-terminal including a three-unit domain of tetratricopeptide repeat motifs that interact with a few proteins [32–35]. FKBP1 regulates the response to corticosteroids which are the most effective controllers of asthma [16]. Sura et al. [16] exhibited the association of FKBP5 polymorphism with asthma susceptibility in patients with asthma. Furthermore, evidence revealed that airway dysbiosis was linked to clinical characteristics in asthma and bacterial microbiota were correlated with specific airway epithelial gene expression signatures [36]. FKBP5 gene expression is associated with Actinobacteria [36].

Zinc finger and BTB domain containing 16 (ZBTB16) encodes a transcription factor [37]. Many biological processes are associated with ZBTB16 including stem cell maintenance and proliferation, spermatogenesis, hematopoiesis, metabolism, and immunity [38, 39]. The correlation between ZBTB16 and asthma is embodied in the treatment of asthma using inhaled glucocorticoids or corticosteroids (ICS), which was indicated in the study [40] that increased expression of ZBTB16 could reduce inflammatory signaling and gene expression, which could contribute to the therapeutic efficacy of ICS.

The wingless-integrase-1 (WNT) signaling pathways, composed of a family of secreted glycoproteins [41–43], regulate various process fundamental to normal development, including cell proliferation, polarity, differentiation, adhesion and motility [18, 44, 45]. WNT5A is a non-canonical WNT ligand that is highly evolutionary conserved [18]. Previous studies have illustrated that WNT5A is utilized the airway remodeling on multiple levels [41]. Extracellular matrix turnover is increased in human airway smooth muscle (ASM) by WNT5A via functional interactions with TGF-β [46]. In addition, therapy of human ASM with recombinant WNT5A enhances formation and contractility of actin filaments [47]. Notably, WNT5A expression in bronchial biopsies is linked to Th2-high asthma [48]. Wnt inhibitory factor-1(WIF1) is a Wnt antagonist and tumor suppressor [49]. Wang et al. [50] concluded that WIF1 was associated with lung function and participating in inflammatory pathways exerts an effect on the level of lung function.

## Conclusion

Combining the gene expressed microarrays and gene methylation microarrays via integrated bioinformatics analysis tools, several hub genes and related pathways as well as candidate genes associated with asthma were screened, which may provide new insights to uncover the underlying molecular mechanisms, exploring the novel clues for drug development and develop optimal biomarkers for the precise diagnosis and treatment of asthma. Nevertheless, the specimen size is small. Further studies and experiments will be imperative to confirm these genes and pathways that are connected with asthma.

## Data Availability

The datasets used and/or analysed during the current study are available from the corresponding author on reasonable request.

## Abbreviations

DMGs: aberrantly methylated genes
AUC: area under curve
CC: cellular component
DEGs: differentially expressed genes
FDR: false discovery rate
FKBP5: FK506-binding protein 5
FC: fold change
GEO: Gene expression omnibus
GO: Gene ontology
GSEA: Gene set enrichment analysis
ICS: inhaled glucocorticoids or corticosteroids
KEGG: Kyoto encyclopedia of genes and genomes
MAM: mitochondria-associated ER membrane
MF: molecular function
PPI: protein-protein interaction
PDK4: pyruvate dehydrogenase kinase 4
qRT-PCR: quantitative real-time polymerase chain reaction
WNT: The wingless-integrase-1
WIF1: Wnt inhibitory factor-1
ZBTB16: Zinc finger and BTB domain containing 16.

## References

[1] Li Q, Li HX, Wang MF (2020). Bioinformatics analysis of gene expression profile of upper airway in asthmatic patients. J Hubei Univ Med.

[2] Mims JW (2015). Asthma: definitions and pathophysiology. Int forum allergy rhinology.

[3] Dharmage SC, Perret JL, Custovic A (2019). Epidemiology of asthma in children and adults. Front Pead.

[4] Eder W, Ege MJ, vM E (2006). The asthma epidemic. N Engl J Med.

[5] BA H, XP Y, DK H, JT PZYZ (2020). Identification of candidate aberrantly methylated and differentially expressed genes in esophageal squamous cell carcinoma. Sci Rep.

[6] Li-ping W, Sha L, Yan S, Chong B (2017). DNA methylation and asthma: recent progress. Acad J Sec Mil Med Univ.

[7] Salam MT (2014). Asthma epigenetics. Adv Exp Med Biol.

[8] Karmaus W, Ziyab AH, Everson T, Holloway JW (2013). Epigenetic mechanisms and models in the origins of asthma. Curr Opin Allergy Clin Immunol.

[9] Zhang MY, Ren W, Chen SS, Zhang Q, Li CX, Wan JX (2021). Exploring and bioinformatics analysis of differentially expressed genes in bronchial asthma. Zhonghua yi xue za zhi.

[10] Mullassery D, Smith NP (2015). Lung development. Semin Pediatr Surg.

[11] Herriges M, Morrisey EE (2014). Lung development: orchestrating the generation and regeneration of a complex organ. Development.

[12] Moon SM, Gu H, Ryu HJ, Kim JJ, Kim HT, Han BG (2005). Identification of four novel HLA-DOA alleles, DOA*010106, DOA*0102, DOA*0103, and DOA*0104 N, by sequence-based typing*. Tissue Antigens.

[13] Yucesoy B, Johnson VJ, Lummus ZL, Kashon ML, Rao M, Bannerman-Thompson H (2014). Genetic variants in the major histocompatibility complex class I and class II genes are associated with diisocyanate-induced asthma. J Occup Environ Med.

[14] Naruse TK, Kawata H, Anzai T, Takashige N, Kagiya M, Nose Y (1999). Limited polymorphism in the HLA-DOA gene. Tissue Antigens.

[15] Xiaobin C, Lisheng W, Jiaxi L, Zhengguang C. Progress in the application of Metabolomics in Children’s bronchial Asthma Research. Jilin J Chin Med. 2020;40 5.

[16] Sf A, Ha A, Jh Y, Hf G (2021). The association of FKBP5 polymorphism with asthma susceptibility in asthmatic patients. J Basic Clin Physiol Pharm.

[17] Xiaojun G, Shuling WANG, KLe (2017). Metabolomics Research on TCM syndrome of Childhood Asthma. Chin Archives Traditional Chin Med.

[18] Asem MS, Buechler S, Wates RB, Miller DL, Stack MS (2016). Wnt5a Signaling in Cancer. Cancers.

[19] Lin CY, Chin CH, Wu HH, Chen SH, Ho CW, Ko MT (2008). Hubba: hub objects analyzer–a framework of interactome hubs identification for network biology. Nucleic Acids Res.

[20] Jeong H, Mason SP, Barabási AL, Oltvai ZN (2001). Lethality and centrality in protein networks. Nature.

[21] Chin CS, Samanta MP (2003). Global snapshot of a protein interaction network-a percolation based approach. Bioinformatics.

[22] Sabidussi G (1966). The centrality index of a graph. Psychometrika.

[23] Guan Q, Tian Y, Zhang Z, Zhang L, Zhao P, Li J (2021). Identification of potential key genes in the Pathogenesis of Chronic Obstructive Pulmonary Disease through Bioinformatics Analysis. Front Genet.

[24] Zhou S, Lu H, Xiong M (2021). Identifying Immune Cell infiltration and effective diagnostic biomarkers in rheumatoid arthritis by Bioinformatics Analysis. Front Immunol.

[25] Ma WQ, Sun XJ, Zhu Y, Liu NF (2020). PDK4 promotes vascular calcification by interfering with autophagic activity and metabolic reprogramming. Cell Death Dis.

[26] Jeong JY, Jeoung NH, Park KG, Lee IK (2012). Transcriptional regulation of pyruvate dehydrogenase kinase. Diabetes metab J.

[27] Lee SJ, Jeong JY, Oh CJ, Park S, Kim JY, Kim HJ (2015). Pyruvate dehydrogenase kinase 4 promotes vascular calcification via SMAD1/5/8 phosphorylation. Sci Rep.

[28] Thoudam T, Ha CM, Leem J, Chanda D, Park JS, Kim HJ (2019). PDK4 augments ER-Mitochondria contact to dampen skeletal muscle insulin signaling during obesity. Diabetes.

[29] Marchi S, Patergnani S, Pinton P (2014). The endoplasmic reticulum-mitochondria connection: one touch, multiple functions. Biochim Biophys Acta.

[30] Hamasaki M, Furuta N, Matsuda A, Nezu A, Yamamoto A, Fujita N (2013). Autophagosomes form at ER-mitochondria contact sites. Nature.

[31] Mostafa MM, Rider CF, Shah S, Traves SL, Gordon PMK, Miller-Larsson A (2019). Glucocorticoid-driven transcriptomes in human airway epithelial cells: commonalities, differences and functional insight from cell lines and primary cells. BMC Med Genomics.

[32] Gao Z, Yu F, Jia H, Ye Z, Yao S (2021). FK506-binding protein 5 promotes the progression of papillary thyroid carcinoma. J Int Med Res.

[33] Baughman G, Wiederrecht GJ, Campbell NF, Martin MM, Bourgeois S (1995). FKBP51, a novel T-cell-specific immunophilin capable of calcineurin inhibition. Mol Cell Biol.

[34] Gallo LI, Lagadari M, Piwien-Pilipuk G, Galigniana MD (2011). The 90-kDa heat-shock protein (Hsp90)-binding immunophilin FKBP51 is a mitochondrial protein that translocates to the nucleus to protect cells against oxidative stress. J Biol Chem.

[35] Pei H, Li L, Fridley BL, Jenkins GD, Kalari KR, Lingle W (2009). FKBP51 affects cancer cell response to chemotherapy by negatively regulating akt. Cancer Cell.

[36] Huang YJ, Nariya S, Harris JM, Lynch SV, Choy DF, Arron JR (2015). The airway microbiome in patients with severe asthma: Associations with disease features and severity. J Allergy Clin Immunol.

[37] U N, Konishi SB, Kondo A, Konopka M, Matsuzaki G. H (2021). Zbtb16 regulates social cognitive behaviors and neocortical development. Transl Psychiatry.

[38] Suliman BA, Xu D, Williams BR (2012). The promyelocytic leukemia zinc finger protein: two decades of molecular oncology. Front Oncol.

[39] Šeda O, Šedová L, Včelák J, Vaňková M, Liška F, Bendlová B (2017). ZBTB16 and metabolic syndrome: a network perspective. Physiol Res.

[40] Leigh R, Mostafa MM, King EM, Rider CF, Shah S, Dumonceaux C (2016). An inhaled dose of budesonide induces genes involved in transcription and signaling in the human airways: enhancement of anti- and proinflammatory effector genes. Pharmacol Res Perspect.

[41] Koopmans T, Hesse L, Nawijn MC, Kumawat K, Menzen MH, Bos ST, Smits R, Bakker ER, Van Den Berge M, Koppelman GH, Guryev V (2020). Smooth-muscle-derived WNT5A augments allergen-induced airway remodelling and Th2 type inflammation. Sci Rep.

[42] Smolich BD, McMahon JA, McMahon AP, Papkoff J (1993). Wnt family proteins are secreted and associated with the cell surface. Mol Biol Cell.

[43] Willert K, Brown JD, Danenberg E, Duncan AW, Weissman IL, Reya T (2003). Wnt proteins are lipid-modified and can act as stem cell growth factors. Nature.

[44] Wend P, Holland JD, Ziebold U, Birchmeier W (2010). Wnt signaling in stem and cancer stem cells. Semin Cell Dev Biol.

[45] van Amerongen R, Nusse R (2009). Towards an integrated view of wnt signaling in development. Development.

[46] Kumawat K, Menzen MH, Bos IS, Baarsma HA, Borger P, Roth M (2013). Noncanonical WNT-5A signaling regulates TGF-β-induced extracellular matrix production by airway smooth muscle cells. FASEB J Off Publ Federation Am Soc Exp Biol.

[47] Koopmans T, Kumawat K, Halayko AJ, Gosens R (2016). Regulation of actin dynamics by WNT-5A: implications for human airway smooth muscle contraction. Sci Rep.

[48] Choy DF, Modrek B, Abbas AR, Kummerfeld S, Clark HF, Wu LC (2011). Gene expression patterns of Th2 inflammation and intercellular communication in asthmatic airways. J Immunol.

[49] Wu J, Fang J, Yang Z, Chen F, Liu J, Wang Y (2012). Wnt inhibitory factor-1 regulates glioblastoma cell cycle and proliferation. J Clin Neurosci Off J Neurosurgical Soc Australasia.

[50] Wang SH, Xu F, Dang HX, Yang L (2013). Genetic variations in the wnt signaling pathway affect lung function in asthma patients. Genet Mol Res: GMR.

